# One year outcomes after small incision lenticule extraction ReLEX in the correction of myopia and myopic astigmatism

**DOI:** 10.1186/s12886-021-02195-9

**Published:** 2021-12-08

**Authors:** Cristina Ariadna Nicula, Dorin Nicula, Sorana D. Bolboacă, Adriana Elena Bulboacă

**Affiliations:** 1 Department of Ophthalmology, “Iuliu Hațieganu” University of Medicine and Pharmacy, Victor Babeș Str., no. 8, 400012 Cluj-Napoca, Romania; 2 Oculens Clinic, Calea Turzii, No. 134-136, 400501 Cluj-Napoca, Romania; 3 Department of Medical Informatics and Biostatistics, “Iuliu Hațieganu” University of Medicine and Pharmacy, Louis Pasteur Str., no. 6, 400349 Cluj-Napoca, Romania; 4 Department of Pathophysiology, “Iuliu Hațieganu” University of Medicine and Pharmacy, Victor Babeș Str., no. 8, 400012 Cluj-Napoca, Romania

**Keywords:** Myopia, Myopic astigmatism, Small Incision Lenticule Extraction (SMILE), Femtosecond laser

## Abstract

**Purpose:**

To report the visual and refractive outcomes of small incision lenticule extraction ReLEX (SMILE) technique using VisuMax femtosecond laser in myopia and myopic astigmatism patients.

**Material and methods:**

A non-randomized clinical study has been conducted on patients with myopia and myopic astigmatism who underwent ReLEX SMILE technique, using the Zeiss VisuMax Laser system (Carl Zeiss Meditec AG, Jena, Germany) at Oculens Clinic, Cluj-Napoca, Romania. Patients older than 18 years, with ocular astigmatism up to -5 diopters (D), spherical equivalent up to -10.00 D, corrected distance visual acuity (CDVA) of 0.3 or better before the surgery, stable refraction for one year, and with a minimum calculated post operator residual stromal bed of 250μ were included in the study.

**Results:**

The study involved a total of 25 myopic eyes (median of sphere diopters equal with -4D) and 67 myopic astigmatic eyes (median of cylinder diopters equal with -1.5 D). The mean refractive spherical equivalent (MRSE) on patients with myopic eyes reduced from -4.25D (median) to -0.5D at one month follow-up, -0.25 D at 6 and 12 months. The mean refractive spherical equivalent (MRSE) on patients with astigmatic myopic eyes reduced from-6.25 D to -0.67 D at one month, -0.62 D at six and twelve months. The value of sphere decreased postoperatively on myopic eyes with a median of -0.25D at one, six and twelve months. The value of cylinder decreased postoperatively on myopic astigmatic eyes with a median of -0.50 D at one month, -0.25 D at six months and -0.50 D at 12 months. At 6 and 12 months, 20 (80.0%) of myopic eyes were maintained within ±0.5 D and 22 (88.0%) with ±1D. On both groups (myopic eyes and myopic astigmatic eyes), statistically significant differences were observed when the keratometric baseline values were compared to each follow-up (*P*-values < 0.0001), without any significant differences between follow-ups (*P*-values>0.15). At 1-month follow-up, uncorrected distance visual acuity (UDVA) was better than or equal to 0.5 in 88.0% of myopic eyes and 82.1% of myopic astigmatic eyes. UDVA remained stable in all cases of myopic eyes at six months and the percentage increased at 92.0% in myopic eyes. UDVA slightly increased at 6-months (85.1%) and remained at the same value at 12-months in myopic astigmatism eyes.

**Conclusions:**

SMILE proved an effective and safe refractive corneal procedure and provided a predictable and stable correction of myopia and myopic astigmatism. SMILE technique demonstrated very good outcomes in terms of keratometric, cylinder, spherical measurements.

## Background

Refractive lenticule extraction (ReLEX) includes small incision lenticule extraction (SMILE) and femtosecond lenticule extraction – the initial form of ReLEX (Flex). ReLEX is an alternative to keratomileusis in situ (LASIK) to correct myopia and myopic astigmatism done in 1990 for the first time by Pallikaris et al. [1]. Moreover, the technique is comparable to femtosecond laser-assisted in situ keratomileusis in terms of efficacy, safety, and predictability [2, 3]. SMILE is becoming more and more popular as a flapless and minimally invasive form of laser vision correction for the treatment of myopia and myopic astigmatism using only one type of laser (femtosecond laser) for the entire surgery. This technology is only available in the VisuMax femtosecond laser platform (Carl Zeiss Meditec AG, Jena, Germany). The intervention with the femtosecond laser platform is minimally invasive and is the latest advancement in laser vision correction of myopia and myopic astigmatism.

Professor Walter Sekundo was the first who performed ReLEX in 2006. ReLEX SMILE is exclusively performed with one laser, a femtosecond laser that ensures high-level reproducibility and predictability, even with high corrections [2–4]. The VisuMax system uses lower pulse energy and higher pulse frequency (500 kHz). A low pulse energy is generally associated with fewer unwanted side effects (such as opaque bubble layer, collateral thermal damage, corneal inflammation) [4]. ReLEX SMILE has the advantages of better ocular surface stability and biomechanical strength compared with femto-LASIK techniques [4–7]. ReLEX SMILE provides a high quality of vision, the reason for being preferred in the treatment of myopia and myopic astigmatism, with an increase in patient satisfaction. During ReLEX SMILE, a VisuMax Femtosecond laser is used to create a disc of tissue called lenticule beneath the anterior surface of the cornea; the lenticule is then extracted through a small opening, eliminating the need for a flap.

Results of several studies reporting the outcomes after SMILE are summarized in Table 1 [8–23].

**Table 1:** Visual and refractive outcomes, safety and predictability of some recent studies performed on small incision lenticule extraction

Study	No. of eyes	Duration, years	Spherical equivalent preop	Spherical equivalent postop	Eyes within ±0.5D	UCVA (%) – ≥20/× postop
Sekundo et al 2011 [8]	91	0.5	-4.75±1.56	-0.01±0.49	80	84 – 20/20
Shah et al 2011 [9]	51	0.5	-4.87±2.16	+0.03±0.30	91	67 – 20/20
Hjortdal et al 2012 [10]	670	0.25	-7.19±1.30	-0.25±0.44	80	61 – 20/20
Karmia et al 2014 [11]	26	0.5	-4.21±1.63	+0.01	100	96 – 20/20
Aǧca et al 2014 [12]	40	1	-4.03±1.61	-0.02±0.06	95	65 – 20/20
Reinstein 2014 [13]	110	1	-2.61±0.54	-0.05±0.36	-	96 – 20/20
Vestergaard 2014 [14]	127	0.25	-7.18±1.57	-0.09±0.45	77	37 – 20/20 73 – 20/25
Pedersen et al 2015 [15]	87	3	-7.30±1.40	-0.40±0.60	70	49 – 20/25
Chan et al 2015 [16]	54	0.17	-6.05±1.46	-0.10±0.23	98.2	76 – 20/25
Wu et al 2016 [17]	39	1	-6.90±0.86	-0.05±0.33	96.7	93 – 20/25
Blum et al 2016 [18]	56	5	-4.89±4.97	-0.375	48.2	n.a.
Yildirim et al 2016 [19]	45	2	-7.10±0.95	-0.30±0.50	92	86 – 20/20
Han et al 2016 [20]	47	2	-6.30±1.47	-0.09±0.39	89	92 – 20/20
Liu et al 2016 [21]	113	0.5	-5.22±1.70	-0.03±0.13	97	96 – 20/20
Ganesh et al 2017 [22]	50	0.25	-0.14±0.28	-0.14±0.28	86	96 – 20/20
Pietila 2018 [23]	300	1	-4.08±1.65	0.04±0.48	91	80 – 20/20

*UCVA* uncorrected visual acuity, *n.a.* not available

Previous studies have demonstrated that SMILE is safe and effective for the management of myopia and myopic astigmatism [13, 14, 16, 22]. The SMILE technique is applied in two centers in Romania, one in Bucharest and the other one in Cluj, at the Oculens Clinique. To the best of our knowledge, no study reporting SMILE safety and effectiveness on the Romanian population has been published in the scientific literature.

We aimed to report the visual and refractive outcomes of ReLEX Smile technique using VisuMax femtosecond laser in patients with myopia and myopic astigmatism at 12 months follow-up.

## Material and methods

### Subjects

A non-randomized clinical study has been conducted on patients with myopia and myopic astigmatism who underwent ReLEX SMILE technique, using the Zeiss VisuMax Laser system (Carl Zeiss Meditec AG, Jena, Germany) at Oculens Clinic, Cluj-Napoca, Romania. All patients with ocular intervention from September 2018 to December 2018 were eligible for the study. Data were retrospectively collected in January 2020 from the medical charts after approval of the study by the ethics Oculens Clinic Ethics Committee (IRB 2018-004). The study was conducted by respecting the Declaration of Helsinki and extensive explanations regarding the procedure, possible intraoperative and postoperative complications. Written informed consent for treatment was obtained from all patients.

Patients older than 18 years, with ocular astigmatism up to -5.00 diopters (D), spherical equivalent up to -10.00D, corrected distance visual acuity (CDVA) of 0.3 or better before the surgery, stable refraction for one year, a minimum calculated post operator residual stromal bed of 250μ, minimal preoperative pachymetry of 500 μm were included in the study. No change in the keratometry data, cycloplegic refraction and axial length of the eye for one year were considered stability refractive criteria. Exclusion criteria were unstable refractive error, ectatic corneal disease (such as keratoconus, pellucid marginal degeneration), glaucoma, radial keratotomy or other previous ocular surgery, retinal degenerative disease, ocular trauma, uncontrolled ocular allergic disease, active blepharitis, significant dry eye, history of herpetic keratitis or any systemic disease that could affect wound healing (e.g., diabetes mellitus, systemic immunodeficiency, autoimmune disorders).

### Ocular examination

A complete ocular assessment was performed prior to the laser correction to all patients. The following were collected for each patient included in the study: uncorrected distance visual acuity (UDVA) and corrected distance visual acuity (CDVA) were measured, refractometry (manifest and cycloplegic), keratometry (Topcon autorefracto-kerato-meter, KR 8900), slit lamp exam (Slit Lamp BX 900, Haag-Streit AG), eye fundus examination, intra-ocular pressure by applanation tonometry, ultrasonic pachymetry (Sonomed 300P Pachymeter), corneal tomography (Pentacam® HR Premium; Oculus Optikgerate GmbH, Wetzlar, Germany), scotopic pupil size measurement with a pupilometer, ocular motor balance, dominance testing, fluorescein break-up time (TBUT) assessed with the slit lamp and endothelial cell counting (Konan SP-9000, Hyogo, Japan). Visual acuity was measured in the Snellen system using a projection chart at 5m. Patients were requested not to wear soft contact lenses for two weeks and rigid gas permeable ones for four weeks before evaluation or surgery.

Spherical equivalent refraction, cylinder, UDVA and CDVA, and keratometry values were measured before the surgery (baseline) and at 1, 6 and 12 months after the procedure.

### Surgical technique

For all interventions, the VisuMax femtosecond laser system (Carl Zeiss Meditec, Jena, Germany) was used. The SMILE procedure was performed in the operating room in sterile conditions by the same surgeon. Topical anesthesia was used in all cases with 2-3 drops of oxibuprocaina solution (Benoxi, UNIMED PHARMA LTD, SLOVACIA) for 3 minutes.

Immediately prior to surgery, the lids were scrabbed with povidone-iodine solution (Betadine, EGIS Pharmaceuticals PLC, Budapesta, Hungary). An eyelid speculum was used to keep the eye wide open. A curved interface, which provided approximate alignment with the corneal surface, was used on the VisuMax femtosecond laser platform. The patient's eye was positioned under the curved contact glass of femtosecond laser. The suction was applied when the center of the pupil was centered on the contact lenses. Initially, the posterior surface of the refractive lenticule was created from the periphery to the center of the cornea. The anterior refractive lenticule was created from the center to the periphery and finally, the side cut was created at 12 o'clock position on a surface of 50° length with the cordial length ranging from 2 to 5 mm. After the laser treatment, a special SMILE double dissector (Dukeworth & Kent, code 6-835) was used to break the remaining tissue bridges between the lenticule and stromal cap and those between the lenticule and stromal bed in order to make the lenticule freely extractable. Then the stromal lenticule was extracted with a special microforceps-SMILE lenticule removal (Dukeworth&Kent, code 6-836E). The laser settings used for expert and standard mode are represented in Table 2 and the surgical parameters used during SMILE are given in Table 3.

**Table 2:** The settings of the laser

Parameters	Standard mode
Frequency, kHz	130-135
Energy offset, (1 offset = 5 nJ)	26-27
Spot distance, μm	
Lenticule and cap cuts Lenticule and cap side cuts	4.2-4.3 2
Track distance, μm	
Lenticule and cap cuts Lenticule and cap side cuts	4.2-4.3 2

**Table 3:** Surgical parameters applied in the SMILE technique

Item	Lenticule	Cap
Optical zone, mm	6.5	
Thickness between, μm	15	120-140
Side cut angle, °	90	90
Transition zone, mm	0.10	
Diameter, mm	6.5	7.50
Incision position, °		90
Incision width, mm		3.93

The thickness of the lenticule was chosen according to the number of diopters to be corrected, considering that 15μm was necessary to correct one diopter.

All eyes aimed for the correction of emmetropia. We did not apply any manual compensation for intraoperative cyclotorsion. All patients received at the end of the procedure one drop of Tobradex (Tobramicine and Dexamethasone) (NOVARTIS PHARMA GMBH - GERMANIA).

After the laser treatment, all patients underwent topical antibiotics and steroids (Tobramicine and Dexamethasone eye drops) five times daily for three days (NOVATIS PHARMA GMBH - GERMANIA), four times daily for one month with the tapered dose and artificial tears two times daily for 2 to 3 months. The patients were trained to avoid make-up and swimming for one week following the SMILE procedure. Slit-lamp examination was performed on 1^st^ and 4^th^ day, 1, 6 and 12 months after the surgery. Uncorrected distance visual acuity (UDVA) and corrected distance visual acuity (CDVA), manifest refraction, spherical equivalent (SE) and keratometry values were measured at 1, 6 and 12 months. Corneal topography was performed at 6 and 12 months postoperatively.

### Statistical analysis

All raw data were collected using a Microsoft Excel spreadsheet (Microsoft Corporation, Redmond, WA, USA). The sample was split into two groups, myopic eyes and myopic astigmatic eyes. Collected data were presented as median and IQR (interquartile range defined as the first quartile to the third quartile) if quantitative (one group was with less than 30 eyes) or number (frequency) if qualitative. Differences between baseline and follow-ups were tested with the Friedman test at a significance level with Bonferroni correction (1.25% for baseline, 1-month, 6-months, and 12-months follow-up; 1.67% for safety and efficacy index) followed by a post-hoc analysis using Wilcoxon test. The safety index (SI) was defined as the (postoperative CDVA)/(CDVA at baseline). The efficacy index (EI) was defined as the (postoperative UDVA)/(CDVA at baseline).

The achieved correction versus the attempted refractive correction at 12 months was analysed with a linear regression model under the assumption of a normal distribution of refractive correction at 12 months. The association between the observed achievement (percentage of eyes within ±0.50 D) at 6-months and 12-months was evaluated with Fisher’s exact test at a significance level of 5%. The association analysis between values of CDVA and keratometry measurements was evaluated with Spearman’s rank correlation coefficient at a significance level of 5%, and any correlation coefficient with *P*-values less than 0.05 were considered statistically significant.

Statistica program (v. 13, StatSoft, USA) was used for statistical analysis and all applied tests were two-tail.

## Results

All eligible patients were systematically evaluated till 12 months postoperatively and were included in the analysis. A total of 92 eyes of 50 patients age from 19 to 63 years were evaluated, 25 myopic eyes (sphere diopters: median = -4, IQR = (-4.5 to -3); 11/14 patients with left and right myopic eyes and one patient with a myopic eye and a myopic astigmatic eye) and 67 myopic astigmatic eyes (cylinder diopters: median = -1.5, IQR = (-2.5 to -1.0), MRSE: median = -6.25, IQR = (-7.25 to -4.125); 31/37 with both eyes with myopic astigmatism). At baseline, the mean age of patients included in the analysis was 31.14± 9.68 years (median = 29.5 years, IQR = (24 to 32)), without significant differences of subjects with myopic eyes as compared to those with myopic astigmatic eyes (Mann-Whitney test: Z-statistics = 1.54, *P*-value = 0.1224). The baseline parameters are shown in Table 4. The follow-up period was 12 months and all patients completed all follow-ups (one, six, and twelve months).

**Table 4:** Baseline parameters of the group

Parameter	Value
Gender, %	
Women Men	57.60 42.40
Spheric equivalent, diopter ^a^	
Myopic eyes Myopic astigmatic eyes Statistic (*P*-value)	-4.25 (-5.25 to -3.12) -6.25 (-7.25 to -4.13) 2.97 (0.0031)
Mean Keratometry, diopter ^a^	
Myopic eyes Myopic astigmatic eyes Statistic (*P*-value)	43.50 (42.75 to 44.85) 44.20 (43.10 to 45.00) 0.66 (0.5103)

^a^ values are expressed as the median and interquartile range (Q1 to Q3), where Q1 is the first quartile and Q3 is the third quartile

### Refractive outcomes: stability and predictability

**Fig. 1:**
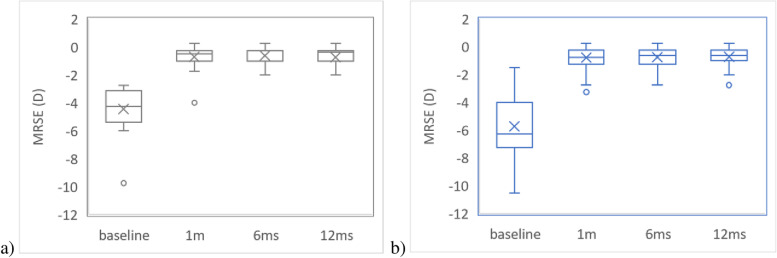
Refractive stability expressed as MRSE (mean refractive spherical equivalent): **a** myopic eyes; **b** myopic astigmatic eyes (m = month)

Refractive stability reproduces the changes in the mean refractive spherical equivalent (MRSE) error after SMILE (Fig. 1). The mean refractive spherical equivalent (MRSE) in patients with myopic eyes reduced from -4.25 D (median) (IQR = (-5.25 to -3.12)) to -0.5 D (IQR = (-0.75 to -0.25)) at 1 month, with a median value of -0.25 D (IQR = (-1 to -0.25)) at 6 months and -0.25 D (IQR = (-1 to -0.25)) at 12 months. Friedman ANOVA showed statistically differences among follow-up examination (Statistics = 72.13, *P*-value < 0.0001), with statistically significance differences when the baseline is compared with each follow-up (*P*-values = 0.00001). Furthermore, no significant differences were observed when 1-month follow-up was compared to 12 months follow-up (Wilcoxon pairs test: Statistics = 1.78, *P*-value = 0.0754) or 6 months follow-up was compared to 12 months follow-up (Wilcoxon pairs test: Statistics = 0.53, *P*-value = 0.5930).

The mean refractive spherical equivalent (MRSE) on patients with astigmatic myopic eyes reduced from -6.25 D (median) (IQR = (-7.25 to -4.13)) to -0.67 D (IQR = (-1 to -0.25)) at 1month, with a median value of -0.62 D (IQR = (-1.13 to -0.25)) at 6 months and -0.62 D (IQR = (-1 to -0.25)) at 12 months. Friedman showed statistically differences among follow-up examination (Statistics = 178.96, *P*-value < 0.0001), with statistically significance differences when the baseline is compared with each follow-up (*P*-values = 0.00001). Furthermore, no significant differences were observed when 1-month follow-up was compared to 12 months follow-up (Wilcoxon pairs test: Statistics = 1.34, *P*-value = 0.1773) or 6 months follow-up was compared to 12 months follow-up (Wilcoxon pairs test: Statistics = 0.53, *P*-value = 0.5930).

The value of sphere decreased postoperatively on myopic eyes with a median of -0.25D (IQR = (-0.75 to 0.00)) at 1 month, -0.25 D (IQR = (-0.50 to -0.25) at 6 months and -0.25 D (IQR = (-0.50 to 0.00) at 12 months. Friedman test showed significant differences among follow-ups (Statistic= 62.02, *P*-value<0.0001). The postoperative mean sphere proved significantly smaller values than baseline for each investigated follow-up (Wilcoxon test: *P*-values=0.00001). Furthermore, no significant differences were identified when paired follow-ups were compared (Wilcoxon test: *P*-values>0.05).

The value of cylinder decreased postoperatively on myopic astigmatic eyes with a median of -0.50D (IQR = (-0.75 to 0.00)) at 1 month, -0.25 D (IQR = (-0.75 to -0.25) at 6 months and -0.50 D (IQR = (-0.75 to 0.00) at 12 months. Friedman test showed significant differences among follow-ups (Statistic = 138.32, *P*-value<0.0001). The postoperative mean sphere proved significantly smaller values than baseline for each investigated follow-up (Wilcoxon test: *P*-values<0.00001). Furthermore, no significant differences were identified when paired follow-ups were compared (Wilcoxon test: *P*-values>0.10), with an identical value of the cylinder at 6 and respectively 12 months for all eyes.

### Predictability

At six months, 20 (80.0%) of myopic eyes were maintained within ±0.5 D and 22 (88.0%) within ±1D. At 12 months 20 (80.0%), the myopic eyes were maintained within ±0.5 D cases and 22 (88.0%) within ±1D. The percentage of eyes within ±0.50 D showed no significant difference between 6 months and 12 months follow-up (Fisher exact test: *P*-value >0.9999). At six months, 29 (43.3%) of myopic astigmatic eyes were maintained within ±0.5 D and 48 (71.6%) within ±1D. At 12 months 29 (43.3%), the myopic astigmatic eyes were maintained within ±0.5 D cases and 49 (73.1%) within ±1D. The percentage of eyes, both myopic and myopic astigmatic eyes, within ±0.50 D showed no significant difference between 6 months and 12 months follow-up (*P*-values >0.9999).

**Fig. 2:**
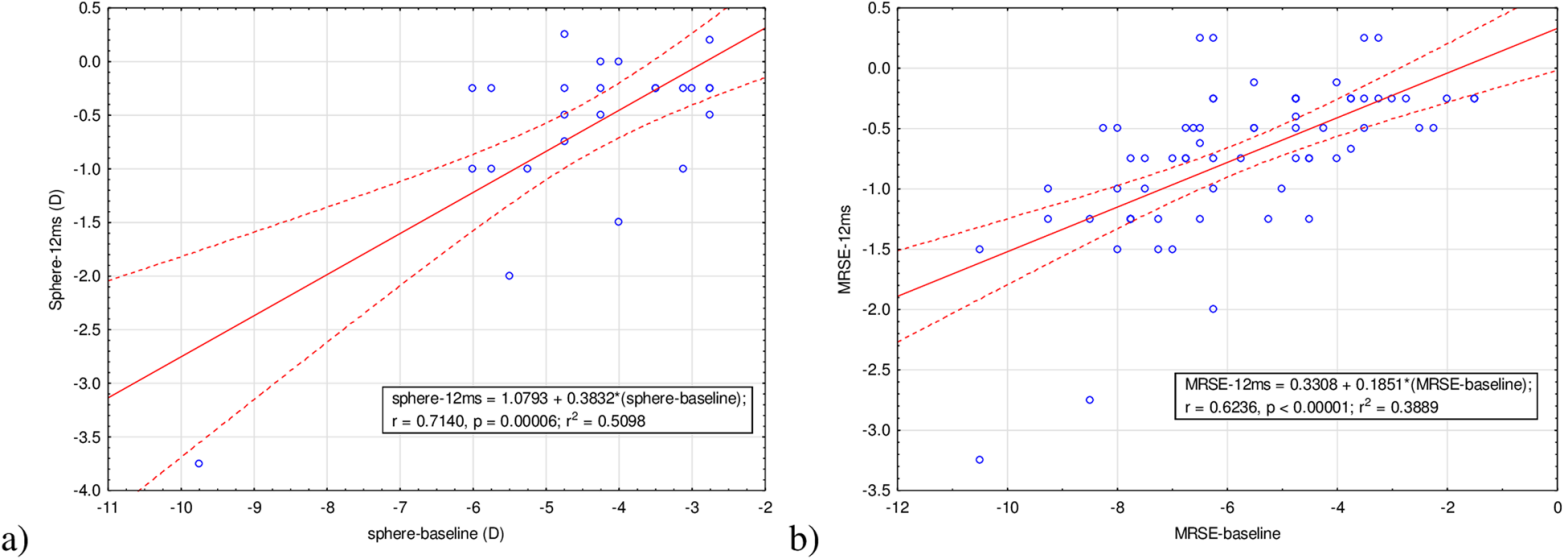
Refractive outcomes of the group at 12 months postoperatively: **a**) attempted versus achieved manifest SE correction in diopters on the myopic eyes; **b**) attempted versus achieved manifest SE correction in diopters on the myopic astigmatic eyes

The association between the achieved and the attempted refractive correction at 12 months after the procedure is presented in Fig. 2.

The Kflat, Ksteep, and Kmean values decreased significantly one month postoperatively and showed similar values at 12 months follow-up on both groups (*P*-values<0.0001). On both groups (myopic eyes and myopic astigmatic eyes), statistically significant differences were observed when the baseline values were compared to each follow-up (Wilcoxon matched test; *P*-values < 0.00002), without any significant differences between follow-ups (Wilcoxon matched test; *P*-value>0.15).

### Functional outcomes: safety and efficacy

All eyes had a CDVA of 0.3 or better at baseline. At 1-month follow-up, UDVA was better than or equal to 0.5 in 88.0% of myopic eyes and 82.1% of myopic astigmatic eyes. UDVA remained stable in all cases of myopic eyes at 6-months, and the percentage increased to 92.0% on myopic eyes. The percentage of UDVA achievement slightly increased at 6-months (85.1%) and remained at the same value at 12-months on myopic astigmatism eyes.

The visual recovery indicates no changes in the UDVA and CDVA between 6-months and 12-months with time after the SMILE surgery, with significantly better 12-months results than baseline (Table 5).

**Table 5:** UDVA and CDVA changes in time: comparisons between baseline and each follow-up

	Myopic eyes ***n***=25		Myopic astigmatic eyes ***n***=67	
	Median (Q1 to Q3)	Stat. (***p***-value)	Median (Q1 to Q3)	Stat. (*P*-value)
UDVA				
Baseline	0.10 (0.10 to 0.20)		0.10 (0.05 to 0.10)	
1 month 6 months 12 months	0.90 (0.60 to 1.00) 0.90 (0.80 to 1.00) 0.90 (0.80 to 1.00)	4.20 (0.00003) 4.20 (0.00003) 4.20 (0.00003)	0.80 (0.50 to 0.90) 0.80 (0.60 to 0.90) 0.80 (0.55 to 0.90)	7.12 (<0.00001) 7.12 (<0.00001) 7.12 (<0.00001)
CDVA				
Baseline	1.00 (0.90 to 1.00)		0.90 (0.70 to 1.00)	
1 month 6 months 12 months	0.90 (0.00 to 1.00) 0.90 (0.00 to 1.00) 0.90 (0.00 to 1.00)	2.75 (0.0060) 2.75 (0.0060) 2.75 (0.0060)	0.40 (0.00 to 0.90) 0.40 (0.00 to 0.90) 0.40 (0.00 to 0.90)	5.06 (<0.00001) 5.05 (<0.00001) 4.95 (<0.00001)

*Q1* first quartile, *Q3* third quartile; Comparisons between baseline and each follow-up was done with Wilcoxon test

The values of the safety and efficacy index are presented in Table 6.

**Table 6:** The safety index and efficacy index expressed as the median and interquartile range (first quartile to the third quartile)

Index	Myopic eyes ***n***=25	Myopic astigmatic eyes ***n***=67	Mann-Whitney test (***P***-value)
Safety index (SI)			
1 month 6 months 12 months	0.90 (0.00 to 1.00) 0.90 (0.00 to 1.00) 0.90 (0.00 to 1.00)	0.71 (0.00 to 1.00) 0.80 (0.00 to 1.00) 0.80 (0.00 to 1.00)	0.59 (0.5536) 0.60 (0.5506) 0.43 (0.6640)
Efficacy index (EI)			
1 month 6 months 12 months	1.00 (0.60 to 1.00) 1.00 (0.80 to 1.00) 1.00 (0.80 to 1.00)	1.00 (0.76 to 1.00) 1.00 (0.83 to 1.00) 1.00 (0.83 to 1.00)	-0.80 (0.4245) -0.95 (0.3432) -0.80 (0.4245)

No significant changes in the safety index was observed in myopic eyes (*P*-value > 0.9999) and in myopic astigmatic eyes (*P*-value = 0.1146). The Friedman test identified significant differences in efficacy index in myopic eyes (Statistic = 12.67, *p* = 0.0018) as well as in myopic astigmatic eyes (Statistic = 20.92, *p*= 0.00003). The significant differences were observed when EI at 1 month was compared to EI at 6-months (Wilcoxon Matched Pairs Test: Statistic = 2.43, *P*-value = 0.0152, *n*=9) and EI at 12-months (Wilcoxon Matched Pairs Test: Statistic = 2.76, *P*-value = 0.0058, *n*=11) on myopic eyes. Similarly, significant differences were observed when EI at 1 month was compared to EI at 6-months (Wilcoxon Matched Pairs Test: Statistic = 3.06, *P*-value = 0.0022, *n*=12) and EI at 12-months (Wilcoxon Matched Pairs Test: Statistic = 3.30, *P*-value = 0.0010, *n*=14) on myopic astigmatic eyes. No significant differences were observed neither on myopic eyes (Wilcoxon Matched Pairs Test: Statistic = 2.02, *P*-value = 0.0431, *n*=5), not on myopic astigmatic eyes (Wilcoxon Matched Pairs Test: Statistic = 0.53, *P*-value = 0.5940, *n*=9) when the EI at 6-months was compared to EI at 12-months.

Positive monotonic association was identified just in myopic eyes between CDVA-6m and Kflat-6m (where 6m = 6-months) - ρ = 0.46 (*P*-value = 0.0212) - and CDVA-6m and Kmean-6m - ρ = 0.45 (*P*-value = 0.0237). Similarly, association between CDVA-12m and Kflat-12m (ρ = 0.46, *P*-value = 0.0212) and CDVA-12m & Kmean 12m (ρ = 0.45, *P*-value = 0.0237) in myopic eyes.

Complications were recorded in 22.82 % eyes, which included a minor epithelial abrasion in ten eyes (9.2%), small tears at the incision in 8 eyes (8.69 %) and some difficulties removing the lenticule in two eyes (2.17%). In one eye, we had a lenticule remnant left in the interface, probably due to incorrect tearing of the lenticule resulting in irregular astigmatism. First of all, we performed an anterior segment ocular coherence tomography to localize the remnant lenticule. After one week, we reoperate, ending with the extraction of the lenticule and visual acuity recovery. No threatening visual complication was recorded and none of the patients showed signs of ectasia at 12 months follow-up. No patient complained about episodes of blurring vision or fluctuation of vision. None of the patients lost lines in CDVA postoperatively.

## Discussions

This is a retrospective, non-randomised study that presented results at 12 months after ReLEX SMILE technique in low and moderate myopia and myopic astigmatism. ReLEX SMILE was used in our study to correct myopia up to -9 D and myopic astigmatism up to -5 D. In our study, we demonstrated a statistically significant decrease of MRSE between baseline and all the follow-ups (*P*-value=0.00001) in myopic and astigmatic myopic eyes. Furthermore, no significant differences were seen comparing the 1 month follow-up with 12 months follow-up. Similar results showed Han et al. [20] in their study of 4 years refractive outcomes, wavefront aberrations and quality of life after SMILE for moderate-to-high myopia (mean SE - 6.30 ± 1.47 D) concluding that SMILE provides a predictable and stable correction, with no significant changes of MRSE during postoperative follow-ups up to 4 years. Similarly, Blum et al [18] reported no significant change of MRSE from a 6-month follow-up to 5-year postoperatively. In the same study, a mild regression of -0.48 D was observed over a period of 5 years [18] as a consequence of eye globe growth rather than a true progression at the corneal level. Wu et al. [17] compared the efficacy and stability of SMILE technique in high myopia versus moderate myopia, concluding that the rate of regression was statistically significant in high myopia compared with low or moderate myopia due to the increased keratocyte activation present in high myopic correction after the procedure.

Our SMILE results reported a high level of refractive predictability. At 12 months, 80% of myopic eyes were within ±0.5D from the target and 88% were within±1 D. At 12 months 43.3%, the myopic astigmatic eyes were maintained within ±0.5 D cases and 73.1% within ±1D. Our results are comparable with refractive predictability reported in the literature [8, 14, 24]. Sekundo et al. [8] reported that 92% of the eyes were within ±0.50D at one year after SMILE procedure. Aǧca et al. [12] showed similar results in 95% of cases. The lower results on myopic eyes in our study than the results reported by Sekundo et al. [8] and Aǧca et al. [12] could be explained by the presence of patients younger than 25 years in our sample on who we performed an extra correction of 0.25-0.5 D. Moshirfar et al. [25] demonstrated that SMILE technique is safe and predictable.

In our study, the safety index at 6 and 12 months was 0.9. Similar results were shown by Ng et al. [24], who obtained a safety index of 1.00 at 3 and 6 months after SMILE and Ivarsen et al. [26] demonstrated a safety index of 1.05 at 3 months after SMILE. Chan et al. [16] in their study of 54 myopic eyes operated with SMILE reported a safety index of 0.97, with 7.4% of cases that lost one line in CDVA.

At one month follow-up, we obtained an UDVA equal or better than 0.5 in 88% of myopic eyes and 82.1% in myopic astigmatic eyes. At 12 months in 92% of myopic eyes and 85.1% of myopic astigmatic eyes, the UDVA was equal or better than 0.6. The efficacy index at all follow-ups was 1 in both groups. Similar results were shown by Pedersen et al. [15], Chan et al. [16] and Wu et al. [17]. Agca et al. [27] showed that the mean postoperative UDVA and CDVA achieved at 5 years of follow-up was 0.04±0.09 logMAR and 0.00±0.04 logMAR, respectively.

The efficacy of SMILE shown above depends significantly on the precision of the lenticule creation by the femtosecond laser. Reinstein *et al* used a very high-frequency digital ultrasound to measure the accuracy of the thickness of the SMILE lenticule and found that the readout central lenticule depth was 8.2 μm thicker on average than the Artemis measured stromal thickness change. This difference was partially explained by alignment errors between the pre- and postoperative scans and partly by central stromal expansion caused by biomechanical changes occurring after SMILE [28].

Regarding important complications, we had a difficult dissection and extraction of the lenticule in two eyes (2.17%) with low myopia. Some studies showed that during the learning curve, this complication was encountered to be present in 16% of cases [29, 30]. To solve the problem is important to identify the correct dissection plane in order to extract the lenticule by using the anterior segment ocular coherence tomography. Meniscus sign (the meniscus-shaped gap between the diameter of lenticule gap and lenticule edge) [31], Shimmer sign (bright reflex around the dissecting instrument) [32] and white ring sign (light reflex from the lenticular side cut) [33] were used to identify the correct dissection plane.

Other complications in our study were recorded in 22.82% eyes, which included a minor epithelial abrasion in ten eyes (9.2%), small tears at the incision in 8 eyes (8.69%). Ivarsen et al. [26], in a study on 1800 eyes, reported an incidence of 6% of epithelial abrasions,1.8% of small incision tears, 1.9% difficult lenticule extraction, 0.22% cap perforation and 0.06% of major tear. In cases of lenticule remnant being left in the interface, Ganesh et al. [34] propose for reevaluation of the anterior segment optical coherence tomography to establish the location of the lenticule. Ganesh et al. [34] concluded that retained pieces of lenticules can be extracted as late as 9 months of the failed SMILE procedure.

In our study, we did not report any ectasia. Moshirfar et al. [25], in their review upon ectasia after 750,000 SMILE procedures performed worldwide, reported only seven eyes with ectasia. The explanation of ectasia was that these cases were undiagnosed fruste keratoconus [35] or had an untrustworthy preoperative corneal topography [36].

In our study, we did not have any cases with severe blurring vision after SMILE. Some studies demonstrated a delay in the visual recovery and visual fluctuations with episodes of blurring of vision in the early postoperative period after SMILE compared to LASIK [37, 38]. This might be as a result of suboptimal laser photo-disruption of the stromal fibers that further causes interface scattering due to traumatic dissection [39], micro-distortions in Bowman’s layer after lenticule removal [40], and transient increased stromal keratocyte activity at the interface resulting from the stromal pocket irrigation [41].

Our study has several limitations that should be acknowledged. This was an observational single center retrospective study, and thus there was no random treatment allocation. However, the inter-physician variability was withdrawn in our study since only one surgeon treated all patients, but we did not control patients’ variability since all eligible subjects were included in the study. The follow-up of our patients’ was limited to one year, but the late follow-up (e.g., 2 years, 5 years, etc.) would also be clinically relevant in the assessment of the long-term effectiveness of this technique. A clinical trial with a large sample (multicenter) and an extended follow-up would confirm the validity of the reported results and the long-term effectiveness. To our knowledge, this is the first study regarding the SMILE technique done in Romania, so our results could be used as input data in sample size calculations on our population.

## Conclusions

SMILE proved an effective and safe refractive procedure and provided a predictable and stable correction of low and moderate myopia and myopic astigmatism. The 12-months results showed that it is a very efficient procedure, with few complications and good visual outcomes. The long-term follow-up would bring clinically relevant information regarding the effectiveness of the ReLEX SMILE technique.

## Data Availability

The raw data analyzed during the current study are not publicly available but are available from the first author on reasonable request.
